# Phenotypic profiling of CFTR modulators in patient-derived respiratory epithelia

**DOI:** 10.1038/s41525-017-0015-6

**Published:** 2017-04-14

**Authors:** Saumel Ahmadi, Zoltan Bozoky, Michelle Di Paola, Sunny Xia, Canhui Li, Amy P. Wong, Leigh Wellhauser, Steven V. Molinski, Wan Ip, Hong Ouyang, Julie Avolio, Julie D. Forman-Kay, Felix Ratjen, Jeremy A. Hirota, Johanna Rommens, Janet Rossant, Tanja Gonska, Theo J. Moraes, Christine E. Bear

**Affiliations:** 1 Department of Physiology, University of Toronto, Toronto, ON Canada; 2 Programme in Molecular Medicine, Research Institute, Hospital for Sick Children, Toronto, ON Canada; 3 Department of Biochemistry, University of Toronto, Toronto, ON Canada; 4 Programme in Developmental and Stem Cell Biology, Research Institute, Hospital for Sick Children, Toronto, ON Canada; 5 Programme in Translational Medicine, Research Institute, Hospital for Sick Children, Toronto, ON Canada; 6 Department of Paediatrics, University of Toronto, Toronto, ON Canada; 7 Firestone Institute for Respiratory Health, Division of Respirology, McMaster University, Hamilton, ON Canada; 8 Department of Molecular Genetics, University of Toronto, Toronto, ON Canada; 9 Programme in Genetics and Genome Biology, Research Institute, The Hospital for Sick Children, Toronto, ON Canada

## Abstract

Pulmonary disease is the major cause of morbidity and mortality in patients with cystic fibrosis, a disease caused by mutations in the *Cystic Fibrosis Transmembrane conductance Regulator (CFTR)* gene. Heterogeneity in *CFTR* genotype–phenotype relationships in affected individuals plus the escalation of drug discovery targeting specific mutations highlights the need to develop robust in vitro platforms with which to stratify therapeutic options using relevant tissue. Toward this goal, we adapted a fluorescence plate reader assay of apical *CFTR*-mediated chloride conductance to enable profiling of a panel of modulators on primary nasal epithelial cultures derived from patients bearing different *CFTR* mutations. This platform faithfully recapitulated patient-specific responses previously observed in the “gold-standard” but relatively low-throughput Ussing chamber. Moreover, using this approach, we identified a novel strategy with which to augment the response to an approved drug in specific patients. In proof of concept studies, we also validated the use of this platform in measuring drug responses in lung cultures differentiated from cystic fibrosis iPS cells. Taken together, we show that this medium throughput assay of *CFTR* activity has the potential to stratify cystic fibrosis patient-specific responses to approved drugs and investigational compounds in vitro in primary and iPS cell-derived airway cultures.

## Cystic fibrosis: toward personalized therapies

A new method for evaluating drug responses in patient-derived respiratory tissue promises to help determine the best treatment for each patient with cystic fibrosis (CF). CF patients are highly susceptible to lung infections due to the build-up of thick mucus in the airways. Over 2000 mutations in the *cystic fibrosis transmembrane conductance regulator* (*CFTR*) gene have been identified in patients with CF, which partly explains their varied response to treatment. Saumel Ahmadi, Christine E. Bear, and colleagues at the Hospital for Sick Children in Toronto developed a fluorescence-based method for measuring improvements in mutant CFTR function in patient-derived nasal and induced pluripotent stem cell-derived lung tissue. This method enables comparison of approved and investigational drugs on airway cells from each individual patient and in the longer term will accelerate the development of personalized therapeutic strategies.

## Introduction

Cystic fibrosis (CF) is caused by mutations in the *Cystic Fibrosis Transmembrane conductance Regulator* (*CFTR*) gene. The CFTR protein functions as a phosphorylation-regulated and nucleotide-regulated anion channel^1–3^ and is localized in the apical membrane of epithelial cells lining the primary conducting airways, intestine, as well as pancreatic, bile, and sweat ducts. Its channel activity provides the driving force for fluid transport and luminal alkalization.^4, 5^ Disease-causing mutations are associated with loss of this function and, in turn, this leads to multi-system pathologies associated with CF including airway infection and inflammation.^6–9^ Pulmonary disease is the major cause of morbidity and mortality in CF patients.^10, 11^

More than 2000 different *CFTR* mutations have been identified in CF patients (www.genet.sickkids.on.ca). Most patients bearing mutations that lead to defects in CFTR channel activation, or “gating mutations”, exhibit a positive response to the “potentiator” called ivacaftor or VX-770, a compound that acts directly to increase phosphorylation-dependent CFTR channel opening.^12–14^ G551D-*CFTR* is one such “gating mutant”, and as it is a rare CF-causing mutation patients bearing this mutation are generally heterozygous with another mutation on the other allele. Although most patients bearing G551D-*CFTR* exhibit improved lung function following treatment with ivacaftor,^15^ approximately 25% fail to show a positive response—a failure that may reflect a number of factors including the influence of the other *CFTR* mutation or other tissue-specific factors.^16^

The major CF-causing mutation, F508del, causes misfolding, misassembly, and mistrafficking of CFTR.^17–19^ In vitro studies in primary airway epithelial cultures obtained at the time of lung transplantation showed that, in combination, the corrector compound lumacaftor (VX-809) plus the potentiator compound ivacaftor were effective in partially rescuing the mistrafficking defect of F508del-CFTR and enhancing its channel activity, respectively.^20–23^ Recently, the combination, ORKAMBI^TM^, was approved by the food and drug administration after Phase III clinical trial data showing that treatment led to modest but significant improvement in lung function. There was a 3–4% increase in Forced Expiratory Volume in the first second (FEV_1_) (ref. 24) in the population of CF patients tested, all homozygous for F508del-CFTR. However, the clinical trial revealed considerable heterogeneity in patient responses with close to 30–40% of patients failing to show a significant increase in FEV_1_ (ref. 25).

Research efforts to discover the next generation of mutation-targeted therapies have escalated rapidly,^26–28^ and in parallel a need to predict patient-specific responses. However, the inaccessibility of in vitro, medium or high-throughput platforms that enables profiling of patient-specific responses to emerging compounds constitutes a major barrier to translation into the clinic. While rectal organoids are being explored as a means to predict patient-specific responses to approved drugs,^29, 30^ such tissues may not fully recapitulate the context of the most severely affected tissue in CF—the airway epithelium. Here, we describe a medium-throughput method that can be applied to study pharmacological modulation of mutant CFTR in patient-derived, primary nasal epithelial cultures and lung derived from induced pluripotent stem cells (iPS cells).

In “proof of concept” studies, we showed that a novel adaptation of a fluorescence-based method for detecting ion channel activity was effective in measuring CFTR activity in primary nasal epithelial cultures and iPS cell-derived lung epithelium grown at the air–liquid interface (ALI)—two new culture systems with the promise of providing a renewable source of relevant tissue for personalized therapy development. Further, an exploratory trial of this in vitro platform revealed its potential impact in defining individuals (homozygous for F508del) with variable responses to the ORKAMBI^TM^ therapy. This exploratory trial also highlighted the potential benefit of introducing a companion therapy to augment the response to ivacaftor in patients who are heterozygous for G551D-CFTR.

## Results

### Fluorescence-based assay of CFTR channel activity in patient-derived respiratory epithelial cultures

The CF drug discovery landscape has recently expanded with the identification of multiple novel modulators of mutant CFTR.^14, 31–33^ Therefore, a priority for the field is to rank the efficacy of emerging compounds relative to approved drugs such as ivacaftor (VX-770) in relevant tissues such as the airway epithelium. Furthermore, given the well-documented variability in drug responses among individuals with an identical *CFTR* genotype,^25^ it is also essential to rank functional rescue by these new compounds in tissues from multiple individuals. Therefore, our primary goal was to develop and test methods for profiling a panel of compounds on patient-specific airway epithelial cultures.

**Fig. 1:**
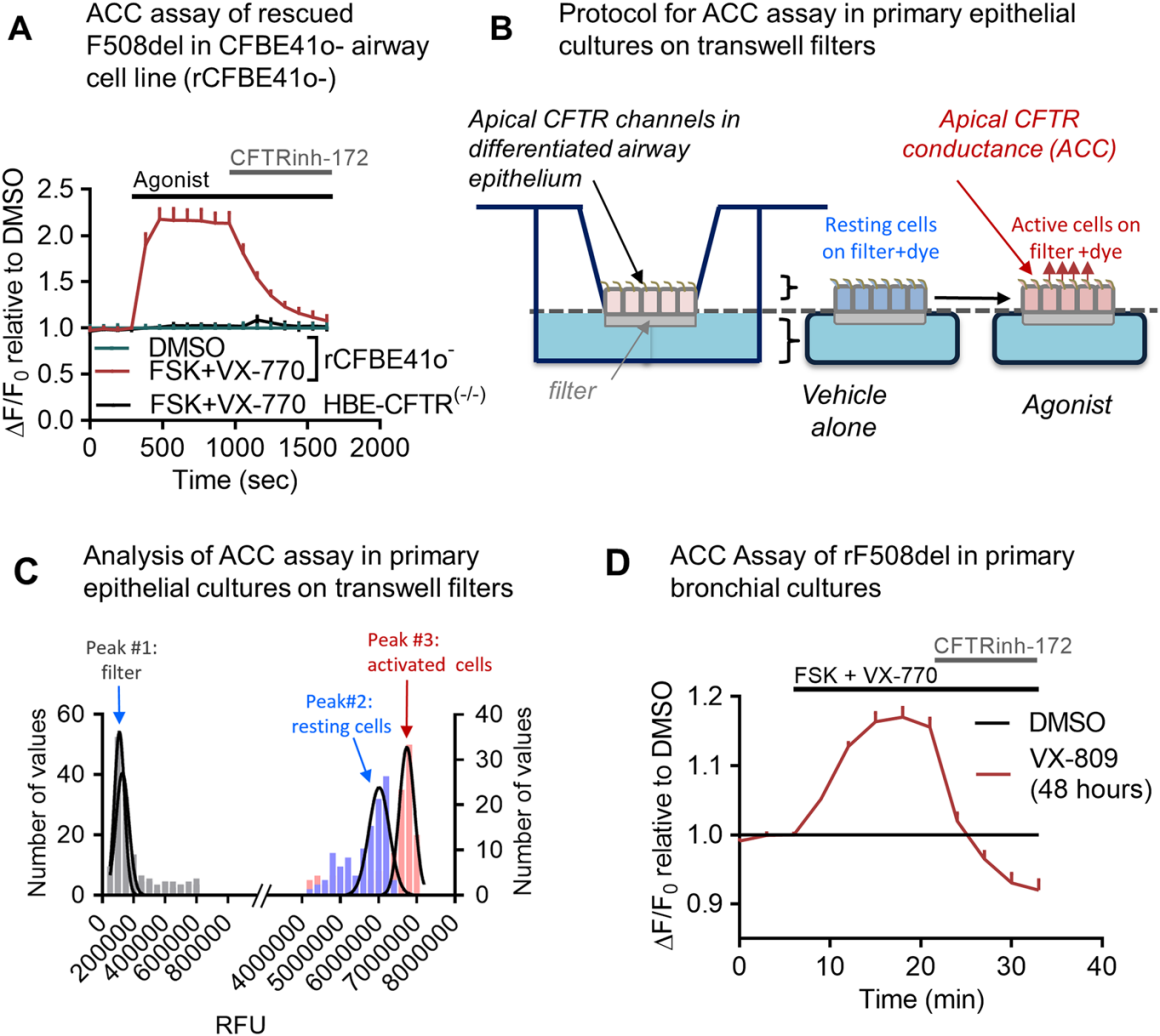
Application of ACC assay to measure F508del-CFTR function in cell lines and primary bronchial tissue. **a** CFBE41o^−^ cells overexpressing F508del-CFTR were rescued with low temperature (27 °C) for 48 h and the fluorescence-based membrane potential assay (ACC) was conducted. F508del-CFTR protein expression after rescue is shown in Supplementary Fig. 2. CFTR activation by FSK (10 µM) and VX-770 (1 µM) caused an increase in chloride conductance leading to membrane depolarization (*red line*), whereas additions of vehicle alone (DMSO) caused a minor deviation (*teal line*). This conductance decreased upon addition of CFTRinh-172 (10 µM), resulting in membrane repolarization. Error bars reflect SD across a 96 well plate (where *n* = 16 individual wells). HBE CFTR knockout cell line is used as a negative control. Disruption of CFTR expression in HBE CFTR(^−/−^) shown in Supplementary Fig. 3. *Black line* represents the effect of CFTR agonist followed by CFTRinh-172 on the HBE CFTR(^−/−^) cell line. **b** *Cartoon* shows components of ACC assay of CFTR-mediated membrane potential changes. Airway epithelial cells, differentiated in ALI on filters in transwell inserts are loaded with membrane potential sensitive dye following application to the apical surface. Resting apical membrane protein is measured in the presence of vehicle, and membrane potential changes mediated by increased ACC determined following the addition of agonist (FSK). **c** All fluorescence pixels from the well are plotted as a histogram and Gaussian curves are fit to values with low and high fluorescence peaks. Peak #1 represents background fluorescence conferred by regions on the filter not populated with living cells, and Peak #2 corresponds to FLIPR® dye intensities conferred by the tissue. The fluorescence corresponding to background is removed by setting a threshold which corresponds to the tail of the Gaussian curve describing Peak #2—or 20% of the maximum fluorescence described by this peak. Peak #3 corresponds to FLIPR® dye fluorescence intensity conferred by CFTR channel activation after agonist addition in the apical membrane of the primary airway cultures increases following activation of CFTR corresponding to Peak #3 (*red*). **d** FLIPR®-based ACC assay and analytical function applied to the study of CFTR activation in primary bronchial epithelial cell cultures from a CF patient. Cultures from this F508del homozygous patient were pretreated with CFTR corrector VX-809 or DMSO control. All cultures were acutely stimulated with CFTR agonist FSK (10 µM) and VX-770 (1 µM), followed by CFTRinh-172 (10 µM). The analytical function (as described in Fig. 1b–c) was applied before calculating mean fluorescence intensity at each time point. The *line graph* represents change in fluorescence relative to baseline (ΔF/F_0_), and this ratio was normalized to the vehicle (DMSO) treated well. The error bars reflect SD (*n* = 3 biological replicates)

The classical method for testing the efficacy of modulators involves measuring the functional expression of mutant CFTR as a cyclic adenosine monophosphate (AMP)-regulated chloride channel in the apical membrane of primary airway epithelial cultures in the Ussing chamber. This method, while providing direct electrophysiological parameters, is low-throughput. The FLIPR® Membrane Potential Plate Reader Assay is commonly used to screen chemical libraries for modulators of normal or mutant ion channels, overexpressed in fibroblast-like HEK-293 cells (Supplementary Fig. 1).^34–36^ We adapted the use of the membrane potential sensitive FLIPR® dye to monitor apical chloride conductance (ACC) mediated by normal and mutant CFTR in airway epithelium. First, we showed that the pharmacological rescue of F508del-CFTR could be measured using the membrane potential sensitive FLIPR® dye in a well-studied CF bronchial epithelial cell line (CFBE41o^−^)^37^ that was modified to overexpress F508del-CFTR.^38^ In these studies, the cells were grown to 5 days post-confluency at low temperature (27 °C for 48 h) to ensure partial rescue of the primary trafficking defect exhibited by F508del-CFTR.^39^ In Supplementary Fig. 2, we show F508del-CFTR protein expression in these rescued cells. In Fig. 1a, we show that apical conduction conferred by F508del-CFTR in rescued (r) CFBE41o^−^ can be detected as membrane depolarization using the FLIPR® membrane potential dye assay after stimulation by forskolin (FSK), an agonist of cyclic AMP-dependent protein kinase A (PKA), and ivacaftor (VX-770), a potentiator of CFTR channel activity. In this study, the sensitivity of this assay was optimized by imposing an outward chloride gradient across the apical membrane. Further, to remove confounding effects of the PKA-sensitive apical sodium channel ENaC,^40, 41^ sodium was replaced with the non-permeant cation, N-methyl-D-glucamine. The CFTR channel inhibitor (CFTRinh-172)^42^ was effective in inhibiting this fluorescence response, supporting the specificity of the depolarization-mediated increase in fluorescence as reporting CFTR channel activity. The specificity of this response for CFTR channel function was confirmed in studies employing a bronchial epithelial cell line in which CFTR was completely disrupted by CRISPR-Cas9 (HBE-CFTR ^(−/−)^). A western blot confirming absence of CFTR in HBE-CFTR ^(−/−)^ cells is shown in Supplementary Fig. 3.

Having optimized conditions for studying CFTR-mediated depolarization in a CF bronchial epithelial cell line, we then developed conditions to measure CFTR-dependent membrane potential changes across the apical membrane of differentiated primary respiratory epithelia obtained from a CF lung transplant patient. In contrast to the epithelial cell lines grown on plastic, such primary airway tissues express endogenous levels of F508del-CFTR, are cultured on a semipermeable filter in a transwell chamber, and exposed to an ALI (Fig. 1b). While transwells provide the optimal substrate for generating properly differentiated airway cultures, the filter confers significant background fluorescence, necessitating the development of a novel analytical technique to derive the specific signal conferred by CFTR channel opening on the apical membrane or apical CFTR conductance (ACC).

This analytical method involves the implementation of a thresholding function, the principles and application of which are shown in Fig. 1b and c. First, the FLIPR® dye fluorescence intensities associated with the transwell chamber alone vs. fluorescence intensities associated with live primary bronchial epithelial cell cultures can be described as a bimodal distribution and fitted with two Gaussian equations, peak #1 and peak #2, respectively. These two Gaussian functions are clearly separated permitting the definition of a threshold fluorescence intensity value (*x*-axis intercept) beyond which the FLIPR® dye intensities can be attributed to properties of the tissue (peak #2). FLIPR® dye fluorescence intensity conferred by CFTR channel activation in the apical membrane of the primary airway cultures increases following activation of CFTR channel activity—corresponding to peak #3 (*red*). This method enabled better resolution of CFTR-specific function in primary bronchial epithelial cultures derived from a non-CF lung transplant donor (Fig. 1c). Further, as changes in fluorescence with activation (*red peak* in Fig. 1c) are specifically normalized to resting cells (*blue peak* in Fig. 1c), this method reports CFTR function that is normalized and cell number independent (Supplementary Fig. 1). In Fig. 1d, we show the kinetics of F508del-CFTR-mediated changes in the ACC assay after correction with VX-809 for 48 h. The ACC response to FSK activation and potentiation with VX-770 is shown as the red line after normalization to this response in cells pretreated with vehicle (DMSO). As expected for F508del-CFTR channel activation, the peak response is reached within 5–10 min and can subsequently be inhibited by CFTRinh-172. In summary, we developed a fluorescence-based method with the potential to detect potentiation of rescued F508del-CFTR on the apical surface of differentiated airway epithelium grown on transwell filters.

### ACC assay of patient-specific responses in nasal epithelial cultures is accurate and reproducible

**Fig. 2:**
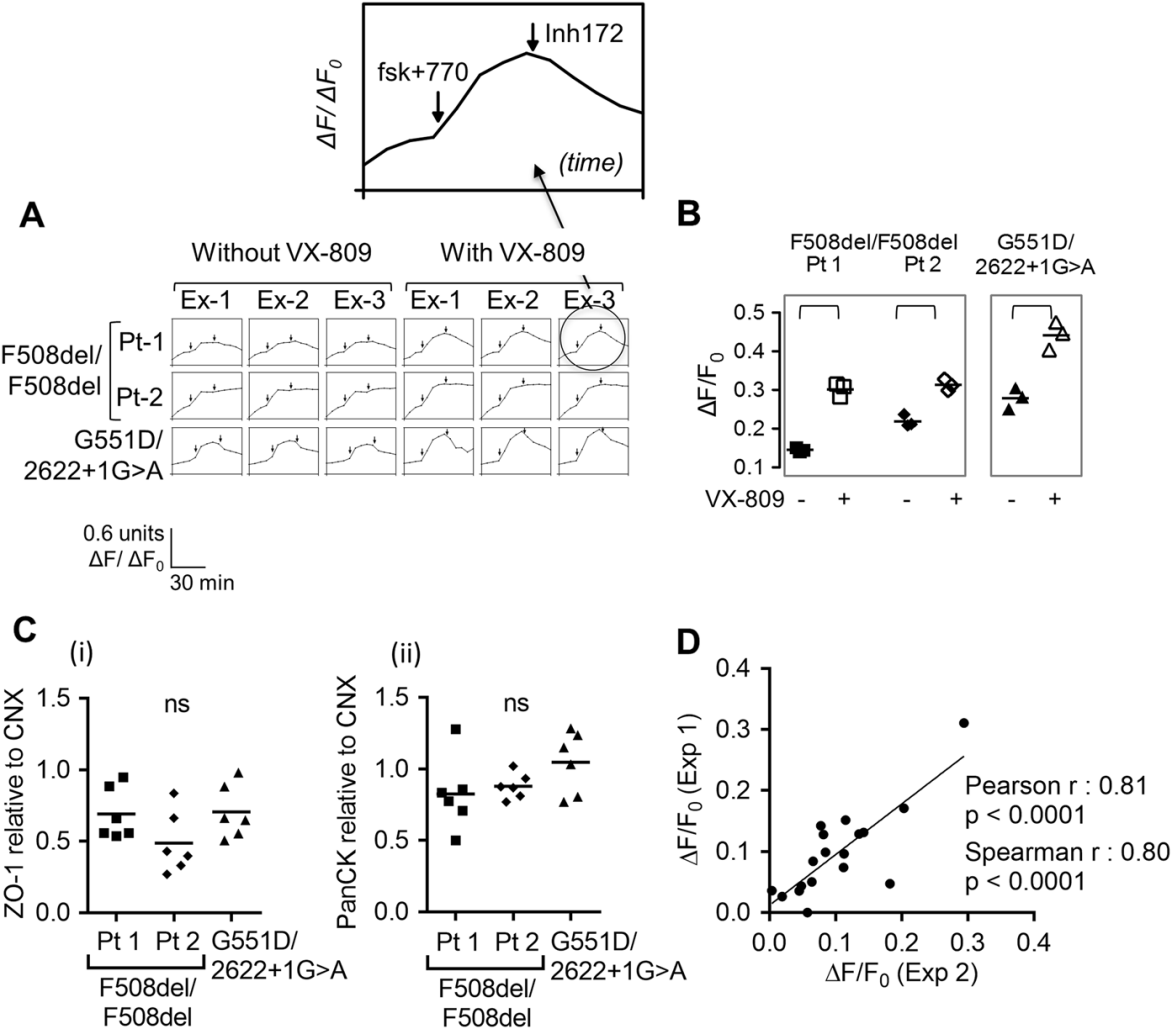
ACC assay is an accurate measure of mutant CFTR function and responses to interventions in patient-specific primary nasal epithelial cultures. **a** Primary nasal cultures from three different CF patients were analyzed in three separate experiments for CFTR function using the ACC assay. Two patients were homozygous for F508del CFTR and one patient had G551D on one allele and 2622+1G>A on the other. Cultures from each patient were pretreated with CFTR corrector VX-809 or control (DMSO) for 48 h. As shown in the magnified well (*upper*, *right*), all cultures were acutely treated with CFTR agonist FSK (10 µM) and VX-770 (1 µM) followed by CFTRinh-172 (10 µM). **b** Scatter plot represents maximum percentage change in fluorescence (ΔF) after addition of CFTR agonist and potentiator, relative to baseline (F_0_) measurements prior to addition. **c** Consistent expression of epithelial differentiation markers: ZO-1, pan-cytokeratin (PanCK) relative to loading control Calnexin (CNX) in each of the wells (i) Densitometry analyses of bands corresponding to ZO-1, normalized to loading control CNX (western blot shown in Supplementary Fig. 4). One-way ANOVA and Tukey’s multiple comparison tests show no significant (ns) difference in the expression of ZO-1 across the three patients (*n* = 6). (ii) Densitometry analyses for PanCK expression normalized to CNX (blots in Supplementary Fig. 4). One-way ANOVA and Tukey’s multiple comparison tests show no significant (ns) difference in the expression of PanCK across the three patients (*n* = 6). **d** Reproducibility between two biological replicates using the ACC assay is shown. Values derived from each experiment are listed in Supplementary Table 1. There is a significant Pearson and Spearman correlation *r* (*p* < 0.0001, *n* = 4 subjects)

We were then prompted to determine if this new method was accurate in reporting in vitro patient-specific responses to pharmacological interventions in the nasal epithelium, a source of respiratory epithelium that is relatively accessible and of interest for testing patient-specific drug responses. In order to assess accuracy and reproducibility, we compared ACC responses with known interventions in primary nasal cultures derived from three CF patients and differentiated on transwells on three separate occasions (experiments 1–3). Nasal cultures from the F508del homozygous patients were treated for 48 h with the corrector compound: VX-809 or vehicle alone. Each box of Fig. 2a shows the individual ACC trace for a separate culture, at the time at which FSK plus potentiator was added and the time at which CFTRinh-172 was added. The cultures derived from the patient heterozygous for G551D (G551D/2622+1G>A) were similarly treated. Quantitation of epithelial cell marker proteins (ZO-1 and pan-cytokeratin C) showed that the cultures were similarly differentiated (Fig. 2c and Supplementary Fig. 4).

It is apparent from Fig. 2a that the ACC traces are different for each patient, with the stimulation with FSK and VX-770 causing a larger peak response in the G551D/2662+1G>A cultures than the cultures from F508del/F508del (without the corrector: VX-809), as expected. Importantly, the ACC responses measured for each donor were similar for experiments 1 through 3 as shown in Fig. 2b, highlighting the accuracy and reproducibility of this assay. Reproducibility of ACC measurements between two different platings was also observed in a larger number of patient-derived cultures (*n* = 19, Fig. 2d, donor genotype and interventions listed in Supplementary Table 1), pointing to the utility of this method in reporting patient-specific responses to CFTR modulators.

### ACC assay correlates with Ussing chamber measurements of patient-specific responses to interventions

**Fig. 3:**
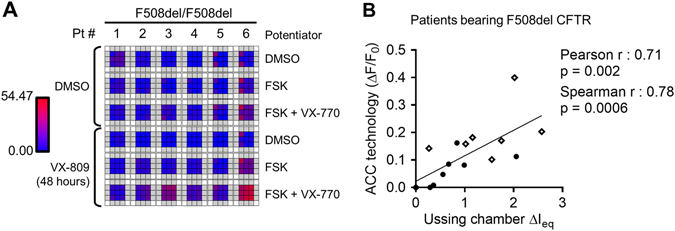
ACC assay correlates with Ussing chamber measurements of patient-specific responses to interventions in primary nasal epithelial cultures. **a** Heat map visualization of apical CFTR chloride conductance measured in nasal epithelia cultured on a 96 transwell plate. Cultures from six F508del homozygous patients subjected to correction—chronic 48 h treatment with VX-809 (or vehicle—DMSO) and acute agonist treatment with FSK and/or VX-770 with DMSO as control. Color scale reflects range of depolarization responses on this plate with *red* representing the maximum response, and *blue* the minimum. **b** Correlation plot of ACC assay and Ussing chamber studies. CFTR-mediated chloride conductance determined in ACC assay (ΔF/F_0_) correlated with CFTR-mediated chloride conductance measured in Ussing chamber studies (ΔI_eq_) for cultures derived from eight patients bearing the F508del mutation. The values derived from each assay for each culture are listed in Supplementary Table 2. A representative Ussing chamber tracing is shown in Supplementary Fig. 5. The diamond-shaped points represent CFTR-mediated FSK responses after correction with VX-809 and potentiation with VX-770 with the filled circles representing FSK responses in the absence of the VX compounds. There is a significant Pearson and Spearman correlation *r* between these assays (*n* = 8 patients, pre-treatment and post-treatment). There was also significant correlation between data acquired using the ACC assay vs. the “Ussing chamber” assay for either pre-treatment or the post-treatment cultures (*n* = 8, *p* = 0.0072, Spearman *r*: 0.88 or *n* = 8, *p* = 0.0279, Spearman *r*: 0.79), respectively

Paired studies of the peak ACC response to FSK and/or VX-770 and peak transepithelial currents as measured in Ussing chambers were conducted in order to assess the correlation between ACC and the gold standard method for assessing functional rescue of F508del-CFTR in tissues derived from patients homozygous for this mutation. As in our previous studies, we focused on studies of patient-derived primary nasal epithelial cultures. In this study, nasal cultures from six different F508del/F508del patients were plated in a 96 transwell plate format for the ACC assay. Nine replicate ACC measurements were obtained from each well by imposing a 3 by 3 matrix as shown in Fig. 3a. The peak ACC responses within each well were color-coded according to the attached scale bar, with “cobalt blue” being a low response and “red” the maximum within the plate. As expected, the peak responses to FSK−/+VX-770 were modest in all of the patient-derived nasal cultures unless pretreated with VX-809. In comparative Ussing chamber studies of nasal cultures from the same individuals (a representative tracing shown in Supplementary Fig. 5), we found that there was significant correlation (Fig. 3b, *p* = 0.002) between the magnitude of the CFTR channel activity as measured in the Ussing chamber and measured in the ACC assay.

### ACC assay enables profiling of existing and novel interventions in patient-specific primary nasal epithelial cultures

We generated a 96 transwell array of nasal cultures from multiple subjects in order to evaluate the potential of our new method to compare patient-specific drug responses and to profile different modulatory compounds.

**Fig. 4:**
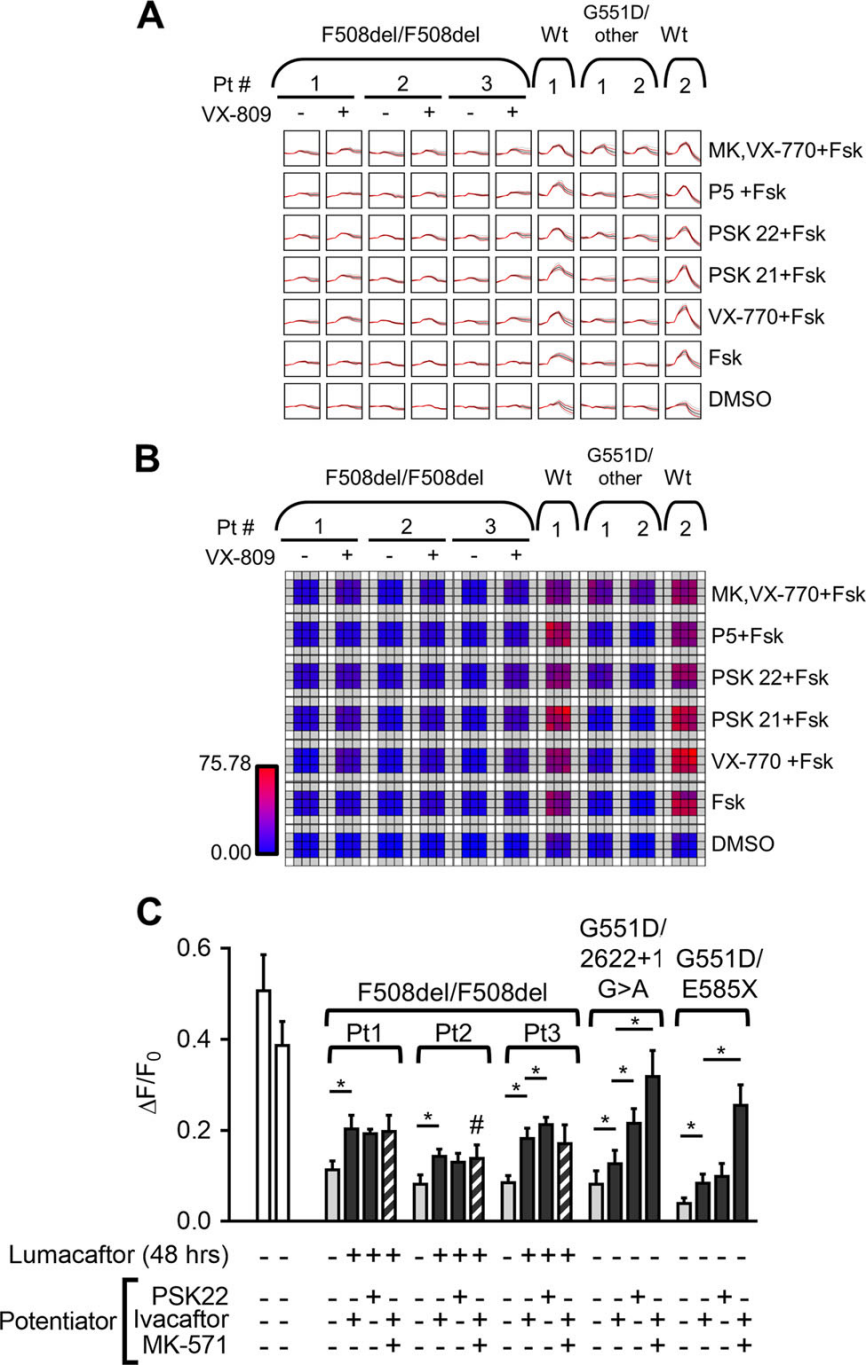
ACC assay enables profiling of existing and novel interventions in patient-specific primary nasal epithelial cultures. **a** Nasal brushings from five CF and two non-CF patients were differentiated together in a 96 transwell plate. Patients bearing the F508del mutation on both alleles were tested after rescue (48 h treatment) with corrector VX-809 or vehicle. During the assay CFTR was activated with FSK (10 µM) plus or minus compounds/drugs listed on the right axis with vehicle (DMSO) as control. CFTRinh-172 was added to terminate the response and assess CFTR specificity. Changes in fluorescence in response to CFTR modulators were monitored over time and simultaneously in multiple regions in each well (*n* = 9). Hence, nine overlapping traces were depicted in each rectangle (magnification shown in Supplementary Fig. 6). Supplementary Figure 7 shows that there is consistent CFTR mRNA expression among the wells and the protein expression of markers of epithelial differentiation is consistent. **b** The data in panel **a** were also represented in the form of a heat map. *Blue* and *red* represents minimal and maximal CFTR stimulations after FSK+/− potentiator for the plate. **c** Bar graphs show peak FSK-mediated responses for nasal cultures from non-CF individuals and peak FSK responses+/− modulators for cultures from CF subjects. Error bars represent SD. Differences were assessed using two-way ANOVA followed by a multiple comparison test. *Asterisks* represent statistical differences of *p* < 0.01. Bars with *white hatched lines* represent responses to ORKAMBI^TM^ for each of the F508del homozygous patients, and differences were analyzed using two-way ANOVA with multiple comparison test. The *hash* sign represents statistical difference (*p* < 0.02) in response to ORKAMBI^TM^ in patient 2 compared with patients 1 and 3

Figure 4a shows the ACC responses to interventions tested on nasal cultures generated from multiple patients, five CF and two non-CF subjects (one to two columns per subject). Of the CF patients studied, three are homozygous for F508del and two heterozygous for G551D. The traces show experimental data similar to that displayed in Fig. 2a, in which, over time, nasal tissues responded to CFTR channel stimulation with depolarization (upward deflection). Subsequently, repolarization was induced after the addition of CFTRinh-172 (downward deflection). Nine technical replicates for responses within each transwell were generated by subdividing each well according to a 3 by 3 matrix and we provide a “zoomed-in” image of the nine replicate traces in two wells in Supplementary Fig. 6. As for the studies of cultures in a 24 transwell plate shown in Fig. 2, we confirmed that the cell density and CFTR mRNA expression was consistent across the 96 transwell plate (Supplementary Fig. 7).

It is clear from Fig. 4a and b (the color-coded heat map of peak responses for the same plate) that the columns containing differentiated, nasal epithelial cultures from non-CF (Wt) individuals exhibit robust depolarization responses after addition of the CFTR channel activator FSK and/or potentiator drugs (VX-770 and four distinct modulators, to be discussed). On the other hand, for columns containing transwells seeded with nasal epithelial cultures derived from CF patients, the amplitude of the response is less, as expected.

As previously mentioned, nasal cultures from patients homozygous for F508del were rescued with the pharmacological corrector VX-809 or DMSO as control for 48 h prior to measuring CFTR channel function. After rescue, F508del-CFTR was stimulated acutely with FSK with or without VX-770 or other investigational modulators (i.e., PSK21 or PSK22). Both PSK potentiators were derived from in-house screens and P5 was obtained from Cystic Fibrosis Foundation Therapeutics (CFFT) since it had been previously described as a potentiator for various mutant CFTR proteins.^43^ In certain cases, the multidrug resistance protein 4 (MRP4) inhibitor, MK-571, was tested.^44, 45^ Inhibition of MRP4 was previously suggested to prevent cAMP efflux within cellular microdomains containing CFTR and thereby augmenting CFTR phosphorylation when added concurrently with agonists of cAMP.^45^ As seen in the bar graph (Fig. 4c), ACC responses of cultures from Wt-CFTR subjects after stimulation with FSK (*white bars*) were approximately four fold greater than the FSK responses from untreated nasal cultures from CF patients (*light gray bars*). With lumacaftor pretreatment and acute potentiation with ivacaftor and FSK, cultures generated from all three patients homozygous for F508del exhibited a statistically significant response (*p* < 0.01). However, absolute functional responses post-treatment were variable for the three individuals. We found that Pt #2 had a significantly lower response to in vitro ORKAMBI^TM^ treatment compared with Pt #1 and Pt # 3 (*p* < 0.02, Fig. 4c).

Also, the absolute responses to various potentiators after correction with lumacaftor were variable for the three individuals homozygous for F508del. Together with FSK addition, acute addition of PSK22 induced a modest improvement relative to VX-770 in Pt #3 (*p < 0.05 or *p < 0.01, respectively). The MRP4 inhibitor, MK-571, did not augment the effect of correction with VX-809 and potentiation with VX-770 in nasal cultures from any of the F508del homozygous patients. Therefore, this platform revealed patient-specific responses to existing and investigational modulators of F508del-CFTR.

This platform also revealed variable responses by two CF patients heterozygous for G551D to the same panel of compounds (Fig. 4c). PSK22 was effective in enhancing the functional expression of G551D for one patient, (G551D/2622+1G>A, *p < 0.01) relative to ivacaftor, pointing to the value of this platform in ranking patient-specific responses to investigational modulators. Interestingly, addition of MK-571 augmented the potentiation mediated by VX-770 observed in nasal cultures derived from both of these patients. We confirmed that primary nasal epithelial cultures express MRP4 using RT-PCR (Supplementary Fig. 8), supporting the claim that MK-571 is mediating this rescue effect by inhibiting MRP4. This is the first evidence in patient-derived tissues supporting a role for MRP4 inhibition as a therapeutic target in CF patients who are heterozygous for gating mutations such as G551D.

Examination of CFTR protein abundance in the same nasal cultures provides a potential explanation for the differential effect of MK-571 in rescuing the functional expression of CFTR in cultures from patients bearing G551D relative to F508del (Supplementary Fig. 9). The abundance of mature and thus plasma membrane-localized G551D-CFTR protein (C band) is close to that detected in non-CF cultures. On the other hand, the abundance of F508del-CFTR protein is low and the mature form visualized as a diffuse, weak band even following lumacaftor treatment. According to the model first proposed by Naren and colleagues, the MRP4 transporter requires expression within a macromolecular complex with CFTR on the cell surface in order to exert a modulatory effect.^45^ Hence, our platform provides the first evidence that MRP4 may augment ivacaftor responses in patients bearing mutations that do not impair CFTR processing.

### FLIPR®-based ACC assay quantifies CFTR channel activity and pharmacological rescue of F508del-CFTR in iPS cell-derived lung tissue

CF researchers have been encouraged by the recent progress in differentiating patient-derived stem cells (induced pluripotent cells) into CF-affected epithelial tissues as these have the capacity for infinite expansion for patient-specific drug-profiling platforms. Further, in proof of concept studies, CF patient (F508del)-derived iPS cells differentiated into proximal lung have been shown to recapitulate the primary defect in the functional expression of CFTR-mediated chloride channel activity.^46, 47^ Such functional studies have been performed using iodide efflux, patch clamp, or Ussing chamber studies of iPS cell-derived lung epithelium.^29, 46, 48, 49^ To date, none of these functional assays in stem cell-derived tissues have been adapted to the medium-throughput format necessary for patient-specific drug profiling. Hence, we tested the potential of our new method to measure the primary defect caused by F508del and its utility in quantifying the pharmacological rescue of F508del-CFTR in iPS cell-derived lung cultures.

**Fig. 5:**
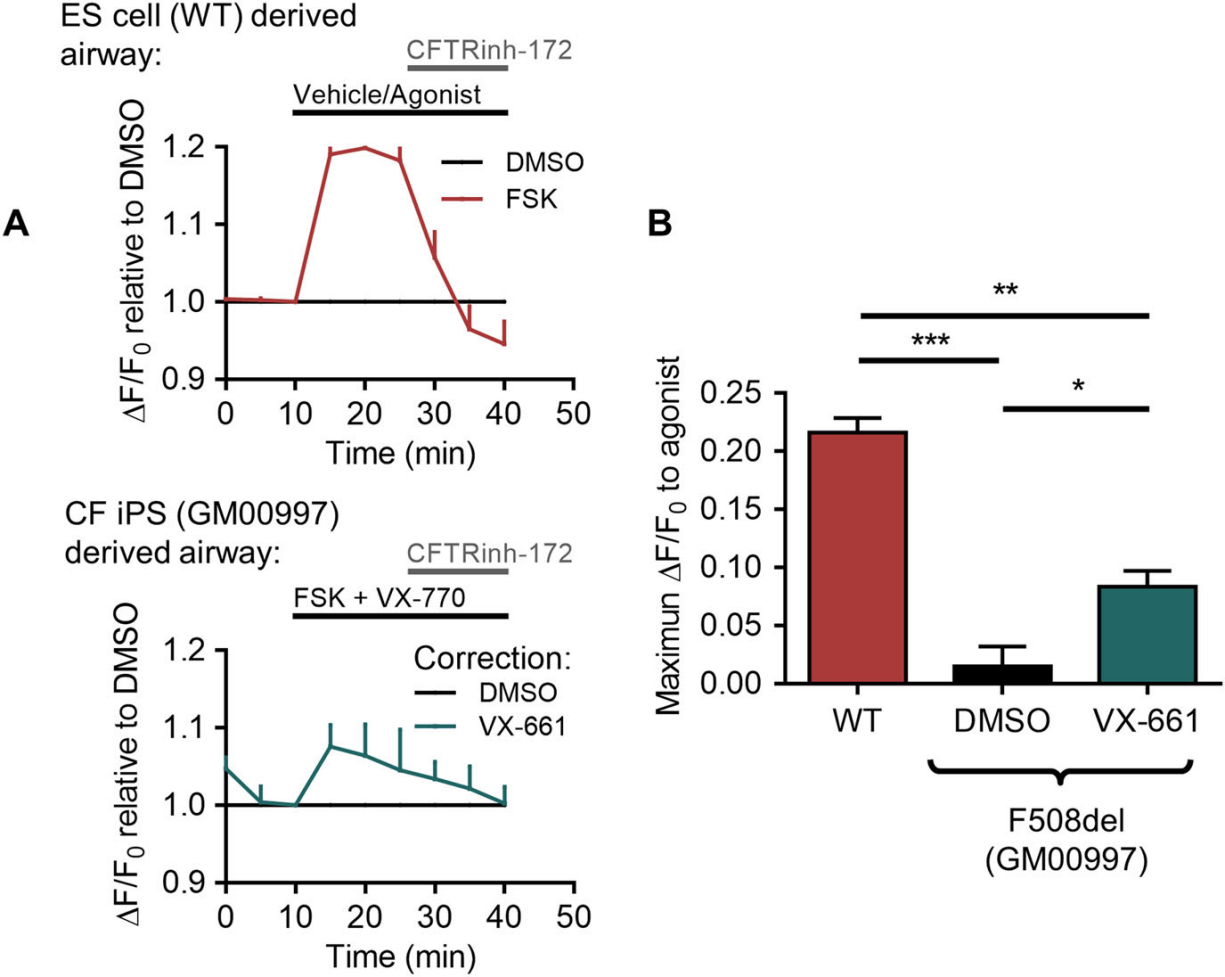
ACC assay reports primary defect and pharmacological rescue of major CF mutant in lung cultures differentiated from iPSCs. **a** ES cell from Wt (CA1) and iPSC derived from F508del CF patient (GM00997) were differentiated to airway epithelia as previously described.^47^ ACC is measured in these epithelia. Airway tissue generated from Wt CA1 ES cells show a robust response to CFTR stimulation by FSK using the fluorescence-based detection method and the analysis is shown in Fig. 1. The changes in fluorescence in activated cultures were normalized to fluorescence measurements in vehicle (DMSO) treated cultures. The traces (*upper panel*) are representative of three biological replicates (or three transwells) wherein >50 regions within each transwell were monitored over time. iPSC-derived airway epithelium from CF-affected individuals were rescued with corrector VX-661 (1 µM) or DMSO as control (*lower panel*). CFTR channels were activated in all cultures by FSK and VX-770 (1 μM). **b** Bar graphs represent maximum percentage change in fluorescence (ΔF) after addition of CFTR agonist, relative to baseline (F_0_) measurements prior to agonist addition (time = 10 min). *Asterisks* indicate statistical significance using one-way ANOVA and Tukey’s multiple comparison tests for the three distinct differentiations (*p < 0.05, **p < 0.01, ***p < 0.001). Immunofluorescence images of CFTR expression in non-CF ES cells and CF iPS cells are shown in Supplementary Fig. 10

We measured the functional response of Wt-CFTR expressed in embryonic stem (ES) cell-derived proximal lung grown on semipermeable supports at ALI using our ACC method (Fig. 5). FSK evoked a robust depolarization (i.e., CFTR activation) in these airway cultures and CFTR inhibition using CFTRinh-172 led to a subsequent repolarization. In contrast, in airway cultures differentiated from iPS cell lines derived from a patient homozygous for F508del-CFTR and corrected with VX-661 (chemically related to VX-809), the FSK response (together with VX-770) was modest, but significant, as shown in Fig. 5. Parallel immunofluorescence studies of the same cultures (Supplementary Fig. 10) confirmed that F508del-CFTR expression was increased after treatment with VX-661. These results recapitulate those previously published using the time-consuming iodide efflux methods using the same iPS cell line^46, 47^ and show the potential utility of the ACC method for studying modulation of mutant CFTR responses in iPS cell-derived lung tissue. Taken together, CFTR function and pharmacological rescue of F508del-CFTR can be assessed using our ACC technique in lung cultures differentiated from patient-specific iPS cells.

## Discussion

Medium-throughput platforms for testing patient tissue-dependent responses to approved drugs and investigational compounds will enhance progress in effectively treating CF, a disease caused by *CFTR* gene mutation and modified by tissue-specific secondary genes. Previously, the feasibility of such *CF clinical trials in a dish* was limited by the paucity of patient-derived lung tissue and the lack of higher-throughput methods to profile modulation of mutant CFTR channel activity by a panel of lead compounds. Recently, the Beekman group showed the utility of functional assays of mutant CFTR-mediated swelling of patient-derived rectal organoids in informing potential treatment strategies.^30^ The fluorescence-based ACC assay described here provides a tool for profiling responses to multiple treatments on patient-derived airway epithelia.

Importantly, we showed that this fluorescence-based method for measuring pharmacological rescue of mutant CFTR is accurate in reproducibly reporting patient-specific responses to CFTR modulators across different platings. It also recapitulates the relative patient-specific responses to treatments observed in the “gold-standard”, but lower throughput Ussing chamber system. In principle, the 24 transwell based, transepithelial chloride conductance (TECC) assay developed by Robert Bridges and colleagues, and used in recent papers by Mutyam et al. and Vu et al.,^50, 51^ measures CFTR channel function in a medium-throughput format. However, to date, there have been no published accounts of the accuracy of the TECC assay in reporting responses by patient-derived tissues to particular interventions across different biological replicates as we show in Fig. 2. Further, unlike the TECC assay, the ACC assay does not require the formation of a tight epithelial resistance as it measures apical membrane potential changes. This difference is particularly relevant in measurements of CFTR function in iPS cell-derived lung cultures, as these cultures are not homogeneously differentiated.^46, 47^

In its current form as a medium-throughout platform, we showed the potential for the ACC assay to identify a novel potential treatment. We showed that the MRP4 inhibitor, MK-571, enhanced the potentiation caused by ivacaftor of nasal epithelial cell cultures derived from CF patients heterozygous for the G551D mutation (i.e., G551D/E585X or G551D/2622+1G>A). Like CFTR, MRP4 is a member of the ATP-binding cassette superfamily of membrane proteins.^44^ It functions as a pump that transports a diverse range of substrates, including lipophilic drugs,^52, 53^ glutathione,^54^ and cyclic AMP.^53, 55^ Hence, augmentation of ivacaftor-mediated potentiation by MK-571 could occur through induction of local elevations in cytosolic cAMP as suggested by Naren and colleagues,^45^ and enhances accumulation of VX-770 or another unknown substrate of MRP4 that modulates CFTR activity. Our platform provided evidence that MRP4 modulation will not be effective in augmenting the response to ORKAMBI^TM^ in airway cultures derived from patients homozygous for the F508del mutation (Fig. 4). This initial observation needs to be confirmed in a larger cohort of patients using this platform.

Our longer-term goal is to optimize the ACC assay such that large chemical libraries can be screened on patient-derived nasal cultures and iPS cell-derived epithelium. To date, such an assay does not exist and there is an urgent need, given the number of rare CF-causing mutations for which there are no therapeutic options. Currently, the Z prime score associated with the ACC assay of CFTR function in a 96 transwell plate of primary nasal epithelial cultures is promising at close to 0.2, but not yet suitable for a robust chemical library screen (Supplementary Fig. 11). The generation of more uniform cultures in the 96 transwell format will constitute a vital step in scaling this assay for high-throughput screens. More efficient protocols are being developed for the differentiation of conditionally reprogrammed nasal cells to differentiated nasal epithelium^56^ or iPS cells to CF lung.^46, 47^ These innovations will enable the generation of uniform cultures of patient-specific tissues on 96 transwell plates—platforms suitable for defining the best intervention for each patient.

Finally, we have yet to test the utility of the ACC assay of patient-derived respiratory tissues in predicting the clinical outcome to existing and emerging therapies. Currently, we are testing the predictive power of this platform in collaboration with clinical scientists as patients (F508del homozygotes) are being enrolled for treatment with ORKAMBI^TM^ and providing drug naïve nasal epithelium and blood samples for iPS cell generation.

## Materials and methods

### CF cell line

The CFBE41o^−^ cell line,^37^ modified to overexpress F508del CFTR,^38^ was used to optimize experimental conditions necessary to detect pharmacological rescue of F508del-CFTR function using the fluorescence-based assay of CFTR-mediated depolarization (see “Membrane potential assay” below).

### iPS cell-derived airway epithelial cell generation

The human ES cell line CA1 was differentiated into lung epithelium as previously described in detail.^46, 47^ In brief, CA1 cells adapted to single cell passage were plated on a 10 cm (2.5 × 10^6^ cells) dish coated with matrigel 24 h prior to the induction of definitive endoderm (DE), according to the manufacturer’s protocol (DE Kit, StemDiff, Vancouver, Canada). DE cells were plated on transwells of a 12 well plate coated with human placenta collagen IV at a density of 5 × 10^5^ cells/well and pushed toward anterior foregut endoderm using high concentrations of FGF2 in the presence of SHH. Differentiation into lung progenitors and immature lung cells were performed as previously described.^47^ The epithelium was polarized using ALI and the cells were used after 3–5 weeks in ALI culture.

### Nasal cell culture method

As described previously,^57^ nasal brushings were performed, and the cells were cultured in basal epithelial growth media (Lonza, Walkersville, MD). After two passages, cells were plated on a collagen-coated 96 well transwells to differentiate them, and basal differentiation media (PneumaCult, StemCell Tech., Vancouver, Canada) was used. After 21 days of growth under ALI, they were used to measure function using the membrane potential assay.

### Western blotting

Cells from transwell or regular multi-well plates were lysed using the modified radioimmunoprecipitation assay buffer (50 mM Tris·HCl, 150 mM NaCl, 1 mM EDTA, pH 7.4, 0.1% (v/v) SDS, and 1% (v/v) Triton X-100) containing a protease inhibitor cocktail (Roche) for 5–10 min. The lysates were then spun down at max speed (>10,000 rpm) for 5 min using a table-top centrifuge. The supernatant was then collected in a separate tube and Laemmli sample buffer was added (1/5 dilution), and then sample was run on a SDS-PAGE gel. The protein from the gel was then transferred to a nitrocellulose membrane and blocked with 5% (w/v) milk. After blocking, the membrane was incubated with one of the following antibodies: human tight junction protein 1 (ZO-1, 1:5000, Life Technologies), human cytokeratin (pan clone AE1/AE3, 1:500, Zymed), human CFTR (UNC 596, CFFT), human Na^+^/K^+^ ATPase (mouse monoclonal a6F, DSHB, Developmental Studies Hybridoma Bank at the University of Iowa), and human calnexin (1:10000, Sigma). After incubation with any of the above, the membrane was incubated with horseradish peroxidase-conjugated secondary antibody raised in goat (against mouse or rabbit primary antibody, 1:2000 dilution), and after multiple washes chemi-luminescence signal was detected using the Li-Cor Odyssey Fc image acquisition system. The images were exported in tag image file format, and analyzed using ImageJ 1.42 Q software (National Institutes of Health).

### Immunofluorescence

Samples were fixed with ice-cold methanol in −20 °C for 15 min. They were then washed and blocked in 4% BSA in PBS for 30 min and incubated with the appropriate primary antibodies against CFTR (Abcam), ZO-1 (Thermo Fisher Scientific, Abcam), Pancytokeratin (Abcam), and DAPI (Thermo Fisher Scientific) overnight at room temperature. Primary antibody was washed away with PBS and samples were incubated with monoclonal or polycolonal secondary antibodies (Life Technologies) for 1 h.^58^ Samples were imaged using Olympus IX81 Quorum Spinning Disk Confocal Microscope and Volocity 6.3.

### qRT-PCR

RNA extraction was performed according to the manufacturer’s protocol (Qiagen Micro or Mini Kit). Briefly, cells were lysed and after RNA extraction concentration was measured using NanoDrop 2000. Only samples with a concentration >100 ng/μL were used, with a 260/280 ratio between 1.8 and 2.1. cDNA synthesis was performed using reverse transcriptase (iSCRIPT cDNA synthesis kit—Biorad) or without reverse transcriptase (negative control). Quantitative real-time PCR was performed using Eva green (Ssofast Evagreen—Biorad) fluorophore in 96 well plates (Biorad). The primers used for amplification are listed in Table 1. Gene expression was normalized to GAPDH.

**Table 1:** Primers employed for qRT-PCR

Gene	Forward	Reverse
1. CFTR	5′‐GCATTTGCTGATTGCACAGT‐3′	5′‐CTGGATGGAATCGTACTGCC‐3′
2. GAPDH	5′‐CAAGAGCACAAGAGGAAGAGAG‐3′	5′‐CTACATGGCAACTGTGAGGAG‐3′
3. FOXJ1	5′‐GAGCGGCGCTTTCAAGAAG‐3′	5′‐GGCCTCGGTATTCACCGTC‐3′
4. MRP4	5′‐GGACAAAGACAACTGGTGTGCC‐3′	5′‐AATGGTTAGCACGGTGCAGTGG‐3′

### Ussing chamber

Nasal epithelial cells were studied in a non-perfused Ussing chamber (Physiologic Instruments, San Diego, CA). The buffer (126 mM NaCl, 24 mM NaHCO_3_, 2.13 mM K_2_HPO_4_, 0.38 mM KH_2_PO_4_, 1 mM MgSO_4_, 1 mM CaCl_2_, and 10 mM glucose) was maintained at pH 7.4 and 37 °C and continuously gassed with 5% CO_2_/95% O_2_ mix.^59^ The transepithelial potential (Vte) was recorded and the baseline resistance (Rte) was measured following repeated, brief short-circuit current pulses (1 µA every 30 sec). The results are presented as equivalent transepithelial current (Ieq), which was calculated using Ohm’s law. CFTR function was determined after inhibition of the epithelial sodium channel (ENaC) with amiloride (100 µM, Spectrum Chemical, Gardena, CA) and cAMP activation with FSK (10 µM, Sigma-Aldrich, USA). CFTR activity was calculated as Ieq difference following CFTR inhibition with CFTRinh-172 (5 µM, EMD Millipore Corp., USA). For drug rescue experiments nasal cell were treated with the corrector VX-809 (6 µM) for 48 h and acutely with the potentiator VX-770 (1 µM).

### Membrane potential assay

Cells were grown on regular 96 well plates or transwell plates (individual transwell—24 well plates, or HTS 96 well plates). Sources and format of plates were as follows: (1) nasal brushings from patients or healthy volunteers and plated on 96 well plate HTS transwells (2) CF iPS cell and non-CF ES cell-derived airway epithelial cells were plated on 24-well individual transwells or 96 well HTS transwell plates.

If the plate consisted the use of a transwell, the basal and apical solutions were kept different. The basal side had Hanks’ buffered solution containing chloride, and the apical solution contained chloride-free buffer (150 mM NMDG-Gluconate, 3 mM KCl, 10 mM HEPES, pH 7.35, osmolarity 300 mOsm).^34, 35^ The blue membrane potential dye (Molecular devices) was loaded in the apical compartment only. However, if the plate was a regular multi-well plate (not a transwell plate), then a single solution of chloride-free buffer (NMDG gluconate buffer) was used, and the blue membrane potential dye was dissolved in it at a concentration of 0.5 mg/mL.^35^ After 40 min of loading the dye at 37 °C, 5% CO_2_ and humidified air, the plate was transferred to the microplate reader (Molecular devices i3 multi-well microplate reader). The microplate reader was set up at least 15 min before the experiment. Briefly, the reader was heated to 37 °C and the settings for fluorescence whole well scan were turned on, with multiple points being read around the center. Upon start of the experiment, baseline reads of at least 3–4 scans were made, followed by addition of drugs (2.5 µL/well). After addition of each drug again 3–4 scans were done. The experiment usually ended by addition of inhibitor (CFTRinh-172 10 μM in this case). Since the membrane potential dye can work bi-directionally, the inhibitor response was prominent, as it caused change in chloride conductance across the apical plasma membrane. Upon completion of experiment, the data were exported in a tab delimited format and analyzed using our analysis platform.

### Chemical reagents

All of the following were dissolved in DMSO to make a 1000 fold concentrated stock solutions—FSK (Enzo Lifesciences); Correctors of CFTR—VX-809, VX-661 (Selleck); Potentitor—VX-770 (Selleck), Inhibitor of CFTR—CFTRinh-172 (CFFT); Calcein AM (Affymetrix eBioscience).

### Statistics

One-way ANOVA with Tukey’s multiple comparison test was performed on all data with more than two data sets for comparison, and SD was calculated using data from biological replicates. Unpaired two-tailed *t*-test was performed on data with two data sets. Kolmogorov–Smirnov test was used to determine normal distribution of data for ACC assay and Ussing chamber studies. Both Pearson and Spearman’s rank correlation tests were performed on Ussing chamber and ACC assays. *P* < 0.05 was considered statistically significant. Statistical analyses were performed using GraphPad Prism 6.01.

## Electronic supplementary material

Supplementary information
