# Supplementary material for: Phenotypic profiling of CFTR modulators in patient-derived respiratory epithelia
Source: NPJ Genom Med. 2017 Apr 14;2:12. doi: 10.1038/s41525-017-0015-6 (PMC5481189; doi:10.1038/s41525-017-0015-6)
Supplement: Supplementary file 1 — Supplementary information [file 41525_2017_15_MOESM1_ESM.pdf]

Supplementary Information:

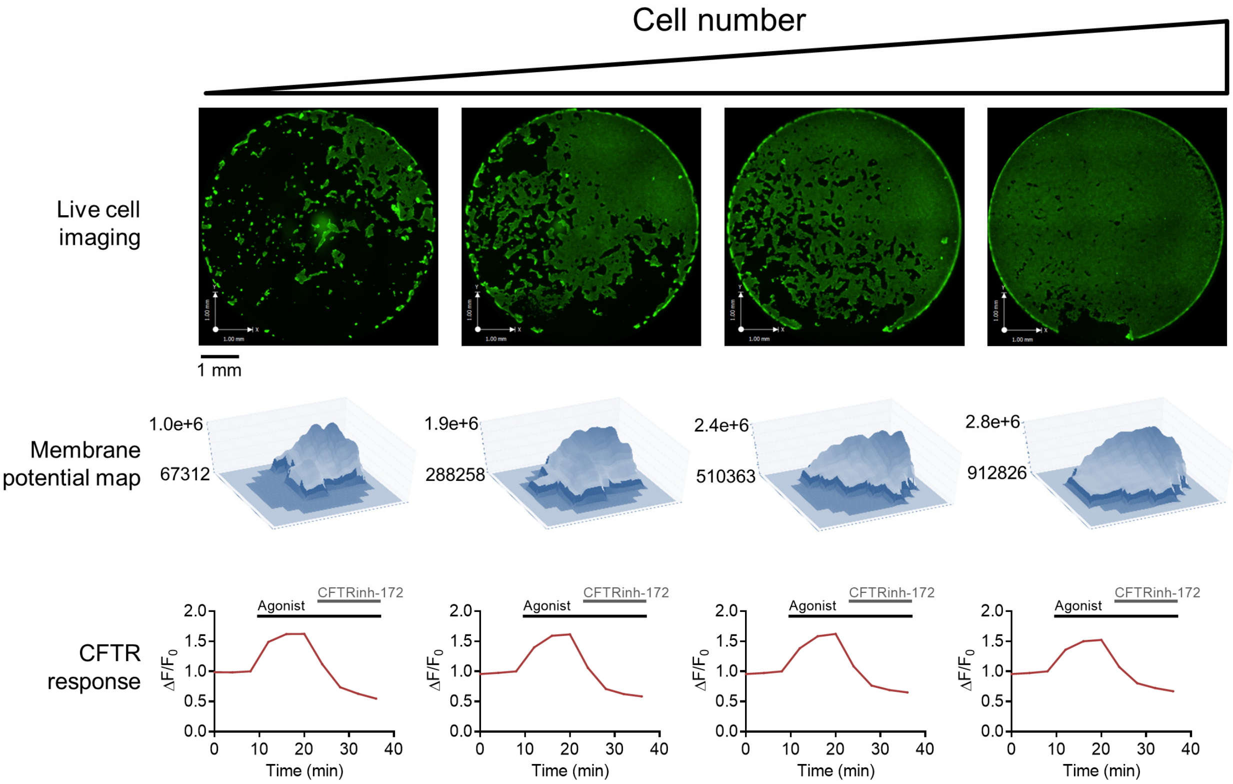

Supplementary figure 1: Detection of Wt CFTR function in HEK293 cells is cell number independent. Increasing number of cells are plated in 4 wells of a 96 well plate. To localize the position of cells in the well, each well is loaded with live cell marker Calcein AM (5 µM) for 30 minutes. Live cell imaging is performed using the epifluorescence microscope. The whole well images obtained from this are shown in the top row. The wells are thereafter loaded with FLIPR® membrane potential dye and a whole well scan is performed. The fluorescence values for each pixel are plotted as a membrane potential map (middle row). The concept of thresholding (described in figure 1) is applied to all the wells, and the mean values for each time-point is obtained. These data are normalized to the last point of the baseline. CFTR is stimulated with cAMP agonist forskolin (10 µM) and thereafter inhibited with CFTRinh-172 (5 µM). Using this method, the CFTR response to forskolin is found to be similar in all the wells irrespective of the cell number.

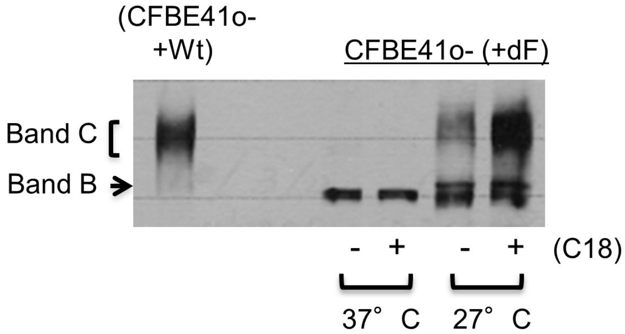

Supplementary figure 2: Rescue of F508del misprocessing in CFBE41o$^-$ cells with low temperature and chemical correction. Whole cell lysates from CFBE41o$^-$ cells overexpressing Wt CFTR, when probed for the CFTR protein on a western blot show both mature Band C and immature Band B forms of the protein (first lane from left). In contrast, CFBE41o$^-$ cells overexpressing F508del CFTR appears only as Band B. However, upon rescue with low temperature both Band C and B are now seen. CFTR specific corrector C18 along with low temperature rescue of F508del CFTR, further increases the mature Band C of the protein (last lane).

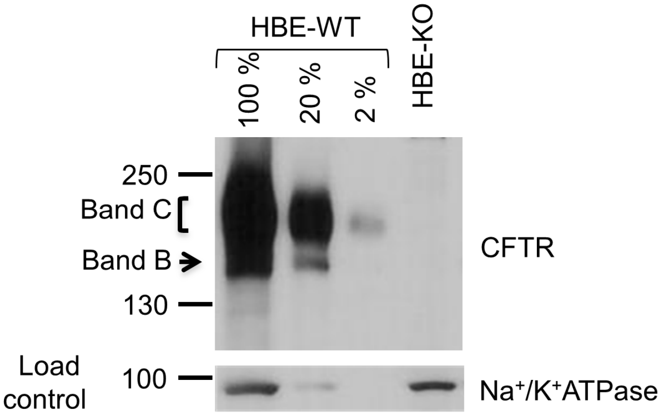

Supplementary figure 3: HBE-knockout cell line lacks CFTR expression. Western blotting of lysates from human bronchial epithelial (HBE) cells, were loaded in decreasing amount (first 3 lanes on the left). Lysate from HBE-KO cell line (CFTR KO), was ran in the last lane. Electrophoretic pattern consistent with Wt CFTR, seen as a lower band B and a higher band C, is seen in the first three lanes (HBE Wt). Both bands are absent in the lysates derived from the HBE-CFTR(-/-) cell line. Na^+/K^+ ATPase was used as loading control.

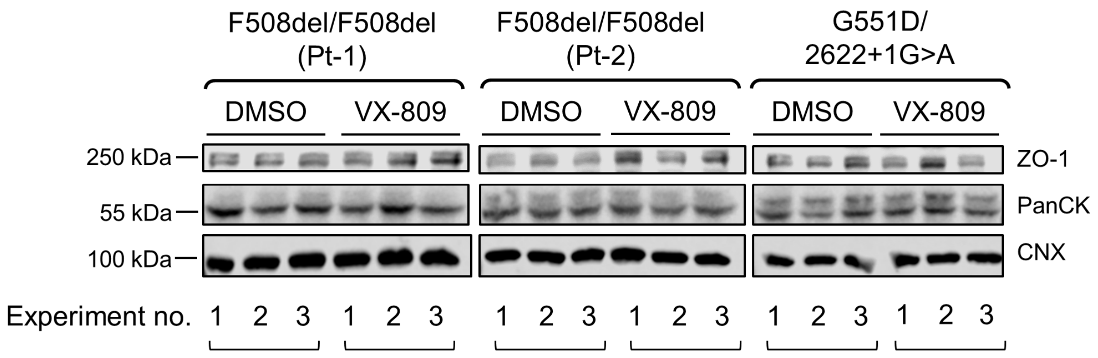

Supplementary figure 4: Nasal cultures from three CF patients have no significant differences in expression of epithelial cell differentiation markers. Nasal epithelial cells from three different CF patients were differentiated to measure CFTR function using the ACC assay (figure 2a). These cultures were tested for markers of epithelial differentiation – ZO-1 and pancytokeratin (PanCK). Analyses of these western blots is shown in Figure 2c, showing no significant differences in these markers across the three CF patients.

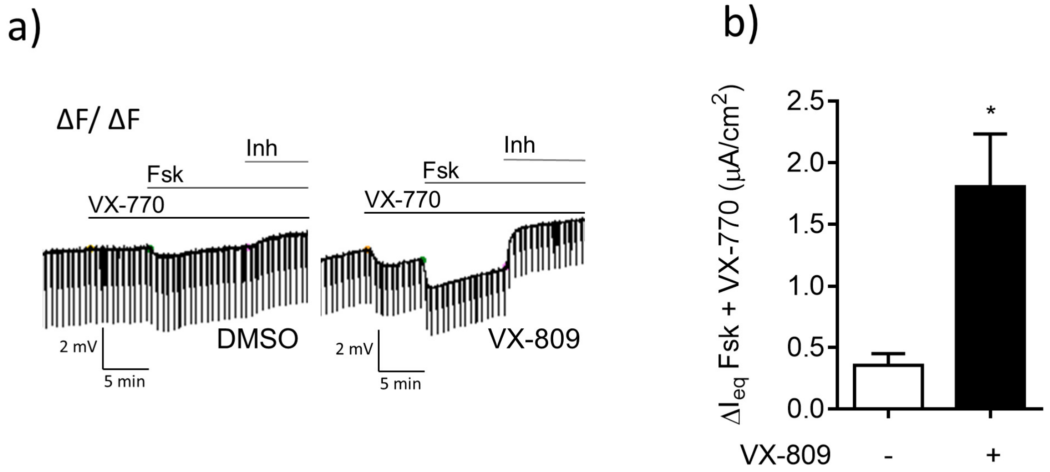

Supplementary figure 5: Representative Ussing chamber studies of bronchial epithelial cultures. (**a**) Bronchial epithelial cells from CF patient homozygous for F508del CFTR were studied using the Ussing chamber. The mutant F508del protein was rescued with either corrector VX-809 or vehicle DMSO. As seen in the figure, acute stimulation of CFTR with potentiator VX-770 (1 μM) followed by cAMP agonist forskolin (10 μM) led to a greater response in the VX-809 rescued cultures compared to vehicle treated. Also, the response to CFTR specific inhibitor CFTRinh-172 was higher after maximal stimulation in the VX-809 rescued cultures compared to control. (**b**) Bar graph represents maximal CFTR stimulation achieved with forskolin and potentiator VX-770, when mutant CFTR is rescued with VX-809 or vehicle alone. Error bars represent SEM from 3 biological replicates. Asterisk indicates statistical significance using unpaired two-tailed t-test (*p = 0.029).

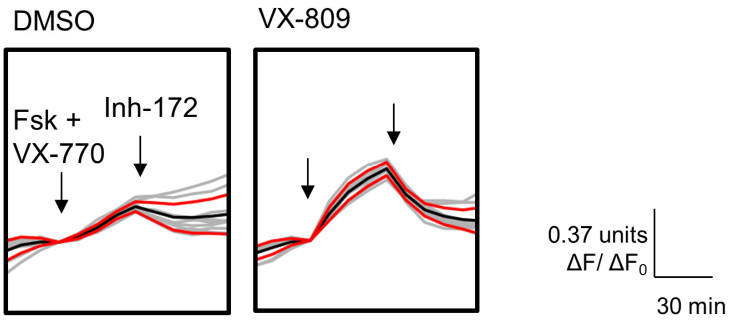

Supplementary figure 6: Representative ACC traces of F508del-CFTR (-/+ VX-809 pretreatment) from nasal epithelial cultures grown on 96 transwell plate. As shown in Figure 4b, each transwell of a 96 well transwell plate was monitored for change in fluorescence in response to CFTR modulators, using the ACC assay. Responses were monitored overtime simultaneously in multiple regions in each well (n=9). The grey lines depict change in fluorescence relative to baseline for each region of the well analyzed. The black line represents mean value at each time point and red lines indicate standard deviation. The line graph shows the response to CFTR agonist forskolin (10 µM) + VX-770 (1 µM) followed by CFTRinh-172 (10 µM). The responses were measured in nasal cultures derived from F508del homozygous patient, rescued with corrector VX-809 or vehicle.

a)

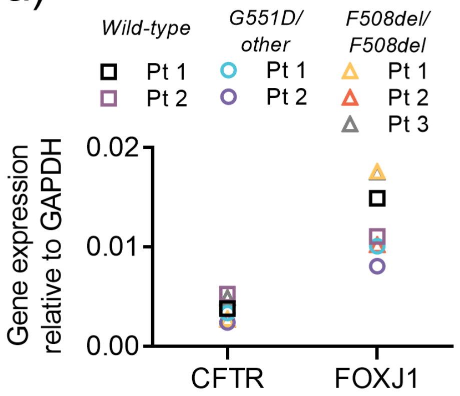

b)

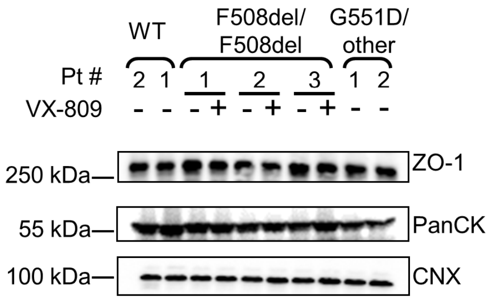

Supplementary figure 7: Nasal cultures grown on 96 transwell plate express epithelial differentiation markers. (**a**) Nasal cultures grown on 96 transwell plate were analyzed for CFTR function using the ACC assay. qRT-PCR studies were performed on them. The expression levels of CFTR, FOXJ1 and SOX17 across all patients was within a 2 fold range, confirming that the cultures were similarly differentiated. (**b**) Western blotting analyses of ZO-1 and pan-cytokeratin (PanCK) protein also showed comparable levels of epithelial differentiation. Calnexin (CNX) analysis assessed protein loading.

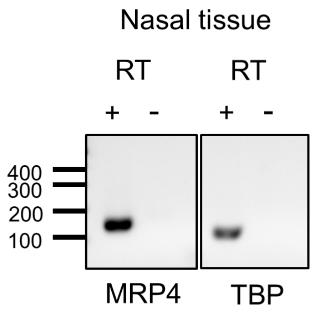

Supplementary figure 8: MRP4 expression in primary nasal cultures. PCR amplification for detecting expression of MRP4 was performed, and it showed that, MRP4 is expressed in differentiated nasal cultures. TBP (TATA binding protein) was used as loading control.

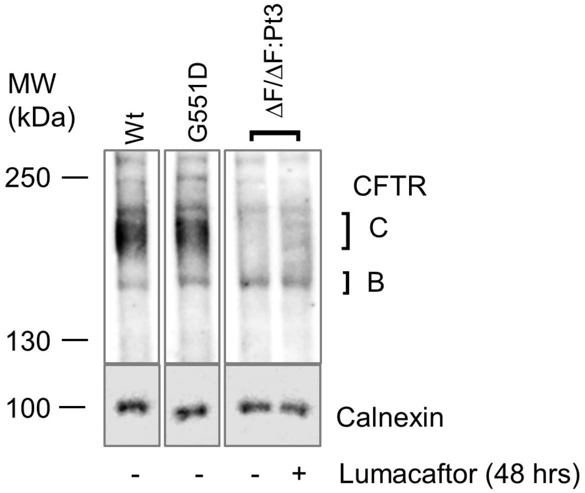

Supplementary figure 9: CFTR protein expression in primary nasal cultures. CFTR protein expression assessed using western blotting of nasal cultures from non-CF (Wt), G551D/2622+1G>A and F508del homozygous patient (rescued with VX-809 or vehicle DMSO). As seen on the blot, mature band C is abundantly present in cultures derived from Wt and G551D subjects and is relatively low in cultures from the F508del homozygous patient, with partial rescue in presence of corrector VX-809.

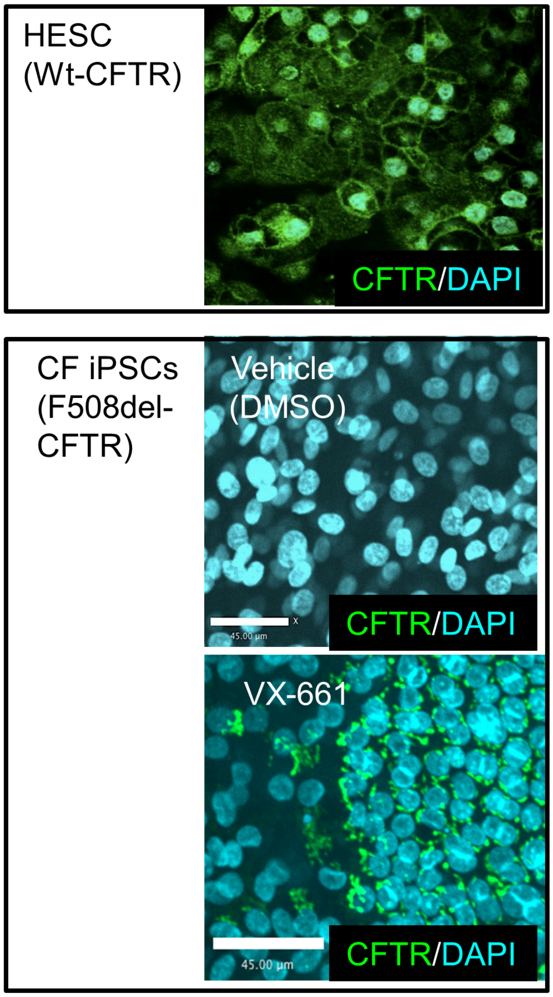

Supplementary figure 10: CFTR and F508del-CFTR localization in airway cultures differentiated from ESC and iPSCs respectively. Immunofluorescence studies of non-CF (Wt) airway cultures derived from CA1 embryonic stem cell (ESC) show that CFTR is expressed on the plasma membrane. Similar studies on airway culture derived from CF iPSC - GM00997 show changes in expression of F508del-CFTR with VX-661 pretreatment, which partially rescues the mutant protein compared to vehicle (DMSO).

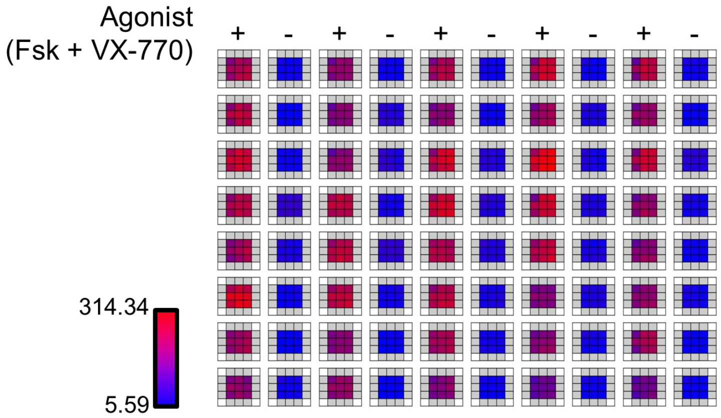

Supplementary figure 11: Z prime test of ACC on 96 transwell plate of nasal epithelial cultures. Nasal epithelial cells derived from a non-CF (Wt) individual were differentiated on a 96 well transwell plate. Each alternating column was stimulated with CFTR agonist Forskolin (10 µM) and VX-770 (1 µM), or vehicle (DMSO) alone. The mean and standard deviation was calculated for both positive control (Forskolin + VX-770 stimulated transwells) and for negative control (DMSO). Calculated Z score was 0.16. There was also a statistically significant difference between negative and positive controls, using unpaired t-test ($p < 0.0001$).

Nasal cultures:

Mutation	Pt no.	Corrector	Stimulant	ΔF/F0 (exp 1)	ΔF/F0 (exp 2)
F508del/F508del	1	DMSO	DMSO	0.057339177	0
		DMSO	Forskolin	0.112906389	0.096223354
		DMSO	Forskolin + VX-770	0.064077511	0.050557535
		VX-809	DMSO	0.048247762	0.043495934
		VX-809	Forskolin	0.115294427	0.15148142
		VX-809	Forskolin + VX-770	0.203293711	0.171103045
	2	DMSO	DMSO	0.019424809	0.026513167
		DMSO	Forskolin	0.081532702	0.128268793
		DMSO	Forskolin + VX-770	0.077734657	0.142197266
		VX-809	DMSO	0.046941835	0.043467138
		VX-809	Forskolin	0.135595083	0.129054993
		VX-809	Forskolin + VX-770	0.142380536	0.13152796
	3	DMSO	DMSO	0.00370574	0.035886526
		DMSO	Forskolin	0.084957324	0.099046931
		DMSO	Forskolin + VX-770	0.066084161	0.084227592
		VX-809	DMSO	0.045011707	0.035470143
		VX-809	Forskolin	0.112055764	0.074065514
		VX-809	Forskolin + VX-770	0.182118595	0.047484502
c.3700A>G/Wt	1	NA	Forskolin	0.2941	0.3107

Supplementary Table 1: Reproducibility of apical CFTR conductance (ACC) assay between two different platings of nasal cultures. Maximum change in fluorescence relative to baseline ($\Delta F/F_0$) from one plating was plotted against the $\Delta F/F_0$ from another plating of nasal cultures derived from the same four different subjects as shown in Figure 5c.

Nasal cultures:

Mutation	Pt no.	Corrector	Stimulant	Δ Ieq	ΔRFU from baseline
F508del/F508del	1	DMSO	Forskolin	0.842	0.1624278
		VX-809	Forskolin + VX-770	2.018	0.3994986
	2	DMSO	Forskolin	0.554	0.04755946
		VX-809	Forskolin + VX-770	1.018	0.1584799
	3	DMSO	Forskolin	2.053	0.1129
		VX-809	Forskolin + VX-770	2.575	0.2033
	4	DMSO	Forskolin	0.989	0.0815
		VX-809	Forskolin + VX-770	0.268	0.1424
	5	DMSO	Forskolin	0.67	0.085
		VX-809	Forskolin + VX-770	1.158	0.1821
	6	DMSO	Forskolin	0.362	0.00852157
		VX-809	Forskolin + VX-770	1.554	0.1017047

Primary airway epithelial cells from lung transplant patients:

F508del/F508del	1	DMSO	Forskolin	0.29	0.0005
		VX-809	Forskolin + VX-770	1.75	0.1704
F508del/G542X	1	DMSO	Forskolin	0.001	0.00043
		VX-809	Forskolin + VX-770	0.001	0.00068

Supplementary Table 2: Correlation of fluorescence based assay of apical CFTR activation (ΔRFU) versus Ussing chamber measurements of CFTR activation (Δ Ieq μA/cm^2) in nasal cultures from the same patients (homozygous for F508del CFTR). Paired measurements were performed for nasal cultures pretreated with VX-809 (3 μM) or vehicle (DMSO) for 48 hours and acutely stimulated with either forskolin alone or forskolin plus VX-770.
